# Supplementary material for: Site-specific length-biomass relationships of arctic arthropod families are critical for accurate ecological inferences
Source: PeerJ. 2023 Sep 6;11:e15943. doi: 10.7717/peerj.15943 (PMC10492534; doi:10.7717/peerj.15943)
Supplement: Supplemental Information 1 [file peerj-11-15943-s001.pdf]

Supporting Information: Article S1

Tom S.L. Versluijs, Mikhail K. Zhemchuzhnikov, Dmitry Kutcherov, Tomas Roslin, Niels Martin Schmidt, Jan. A. van Gils & Jeroen Reneerkens. Site-specific length-biomass relationships of arctic arthropod families are critical for accurate ecological inferences. *PeerJ*

Allometric length-biomass relationships

Dealing with outliers: Before fitting allometric length-biomass regressions we inspected the data for outliers by plotting natural-log transformed biomass against body length. Based on that, we excluded one outlier caused by a measurement error. In addition, we removed 17 datapoints where biomass was not available because of the lower limit of the scales (Mährlein et al. 2016).

Model selection: To be able to compare AIC between models with and without a log-transformed response variable we transformed the AIC value of models with a log-transformed response variable to the arithmetic scale by adding minus twice the logarithm of the Jacobian to the AIC score (Akaike 1978, Burnham and Anderson 2004, Pélabon et al. 2018). Hierarchically more complex models (i.e., nested models with additional parameters) within ΔAIC = 2 of the top-supported model were not considered informative/competitive (Arnold 2010). If multiple models were equally competitive, we preferred the power-model based on residual patterns and biological theoretic foundations (Gowing and Recher 1984, Hayes and Shonkwiler 2006).

Correcting back-transformed data: Fitting linear models using least-squares on log-transformed data ensures minimisation of the sum of the squared residuals on the log-transformed scale (Schoener 1980, Mährlein et al. 2016). This implies that body mass estimates from such

models correspond to the geometric mean instead of the arithmetic mean, resulting in a bias of the estimates towards smaller values (Hayes and Shonkwiler 2006, Mährlein et al. 2016). To account for this bias, we corrected body mass predictions from log-linear models using Duan's smearing factor (Duan 1983). This factor adjusts the estimated body mass on the arithmetic scale by multiplying it with the mean of the back-transformed residuals of the fitted model (Hayes and Shonkwiler 2006, Mährlein et al. 2016). The corrected equation for the power model then becomes $\ln\left(\frac{W}{SF}\right) = \ln(b0) + b1 * \ln(L)$, where the smearing factor is calculated as $SF = \frac{1}{n}\sum_i^n e^{\varepsilon_i}$, where ε_i correspond to the residuals of the fitted model (Mährlein et al. 2016). We refrained from calculating smearing factors when homoscedasticity assumptions were violated. We did not correct for additional biases due to changes in biomass or length as a consequence of preservation in ethanol (Johnston and Cunjak 1999, Mährlein et al. 2016, but see Méthot et al. 2012).

Quantifying model uncertainty: To quantify uncertainty for the fitted allometric equations we calculated 95% quantile confidence intervals for model predictions and regression coefficients using non-parametric (case) bootstrapping using 10,000 bootstrap samples (Efron and Tibshirani 1994, Nakagawa and Cuthill 2007). We opted for non-parametric bootstrapping because normality and homoscedasticity assumptions were violated for several taxa. Although bias-corrected and accelerated bootstrap (BCa) confidence intervals are generally preferred (Efron and Narasimhan 2020), we calculated 95% quantile confidence intervals as the latter resulted in more robust results when dealing with data containing influential datapoints. We only employed bootstrapping for taxa with a sample size of at least n=20.

Calculating biomass: Because two length-biomass regressions are available at Zackenberg for *Chironomidae* (Fig. 1, Table 1), we selected - per specimen - the regression for which the min-max length range of the regression was overlapping with, or was closest to, the length of the specimen.

Assigning body lengths

The Zackenberg pitfall dataset comprises counts of specimens per taxonomic order but does not contain measurements on their length or biomass. Thus, to use our allometric length-biomass regressions for transformation of this dataset from counts to biomass, we first needed to assign body lengths to all specimens. This was done by randomly sampling from taxon-specific length distributions in an additional arthropod dataset (n=42,295 specimens of 54 taxonomic groups) that was collected using pitfall traps and sticky traps at Zackenberg in 2015, 2019 and 2021 and in Knipovich in 2018. All specimens in this dataset were identified to family-level taxonomic resolution, except for Acari and Collembola which were identified to sub-class taxonomic level. Body lengths of all specimens in this dataset were measured under a stereomicroscope to the nearest 0.1 mm from the frons to the tip of the abdomen (excluding any appendages).

References

Akaike, H. 1978. On the likelihood of a time series model. Journal of the Royal Statistical Society. Series D (The Statistician) 27:217–235.

Arnold, T. W. 2010. Uninformative parameters and model selection using Akaike's Information Criterion. Journal of Wildlife Management 74:1175–1178.

Burnham, K. P., and D. R. Anderson. 2004. Multimodel inference: Understanding AIC and BIC in model selection. Sociological Methods and Research 33:261–304.

Duan, N. 1983. Smearing estimate: A nonparametric retransformation method. Journal of the American Statistical Association 78:605–610.

Efron, B., and B. Narasimhan. 2020. The automatic construction of bootstrap confidence intervals. Journal of Computational and Graphical Statistics 29:608–619.

Efron, B., and R. J. Tibshirani. 1994. An introduction to the bootstrap. Chapman & Hall/CRC press, New York.

Gowing, G., and H. F. Recher. 1984. Length-weight relationships for invertebrates from forests in south-eastern New South Wales. Australian Journal of Ecology 9:5–8.

Hayes, J. P., and J. S. Shonkwiler. 2006. Allometry, antilog transformations, and the perils of prediction on the original scale. Physiological and Biochemical Zoology 79:665–674.

Johnston, T. A., and R. A. Cunjak. 1999. Dry mass-length relationships for benthic insects: A review with new data from Catamaran Brook, New Brunswick, Canada. Freshwater Biology 41:653–674.

Mährlein, M., M. Pätzig, M. Brauns, and A. M. Dolman. 2016. Length–mass relationships for lake macroinvertebrates corrected for back-transformation and preservation effects. Hydrobiologia 768:37–50.

Méthot, G., C. Hudon, P. Gagnon, B. Pinel-Alloul, A. Armellin, and A. M. T. Poirier. 2012.

Macroinvertebrate size-mass relationships: How specific should they be? Freshwater Science 31:750–764.

Nakagawa, S., and I. C. Cuthill. 2007. Effect size, confidence interval and statistical significance: A practical guide for biologists. Biological Reviews 82:591–605.

Pélabon, C., M. Tidière, J. F. Lemaître, and J. M. Gaillard. 2018. Modelling allometry: Statistical and biological considerations - a reply to Packard. Biological Journal of the Linnean Society 125:664–671.

Schoener, T. W. 1980. Length-weight regressions in tropical and temperate forest-understory insects. Annals of the Entomological Society of America 73:106–109.
