# Supplementary figures and images for: Site-specific length-biomass relationships of arctic arthropod families are critical for accurate ecological inferences
Source: PeerJ. 2023 Sep 6;11:e15943. doi: 10.7717/peerj.15943 (PMC10492534; doi:10.7717/peerj.15943)

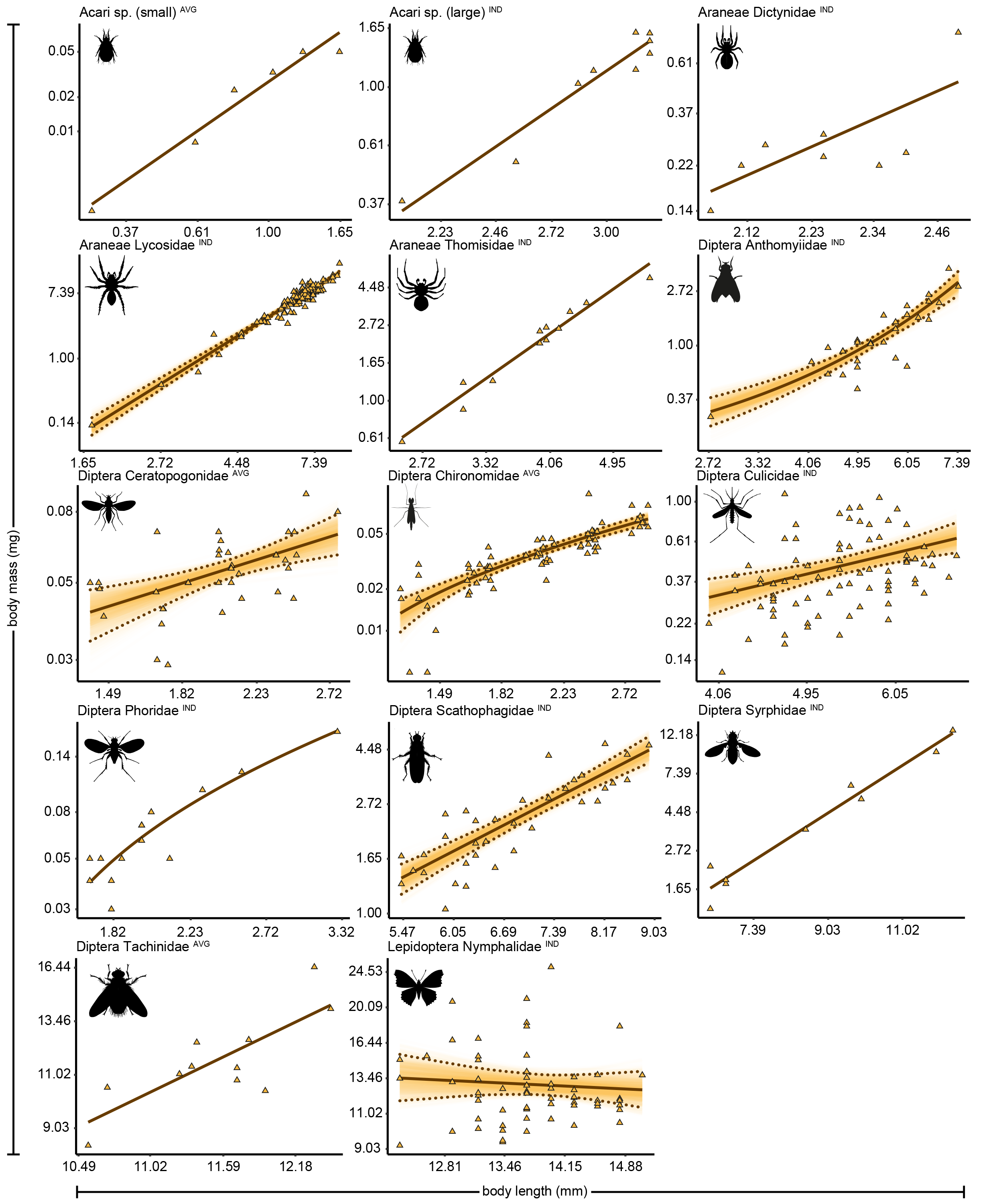

Supplement: Supplemental Information 2 — Axes are log-transformed but labelled with non-transformed values to ease interpretation. Superscripts following taxonomic names indicate whether datapoints represent individual level weight measurements (‘IND’) or averages per length group (‘AVG’). Note that a separate allometric relationship is presented for small Acari for which data are based on averages per length class, and for large Acari for which data are based on individual measurements. Raw datapoints are depicted as orange triangles. Solid lines indicate the best supported model for each taxon. Dotted lines indicate 95% quantile confidence intervals calculated over 10,000 case bootstrapping runs. Individual bootstrapping runs are drawn as transparent lines to create a colour gradient that visualizes the distribution of best fitting models over all bootstrapping runs. [file peerj-11-15943-s002.png]

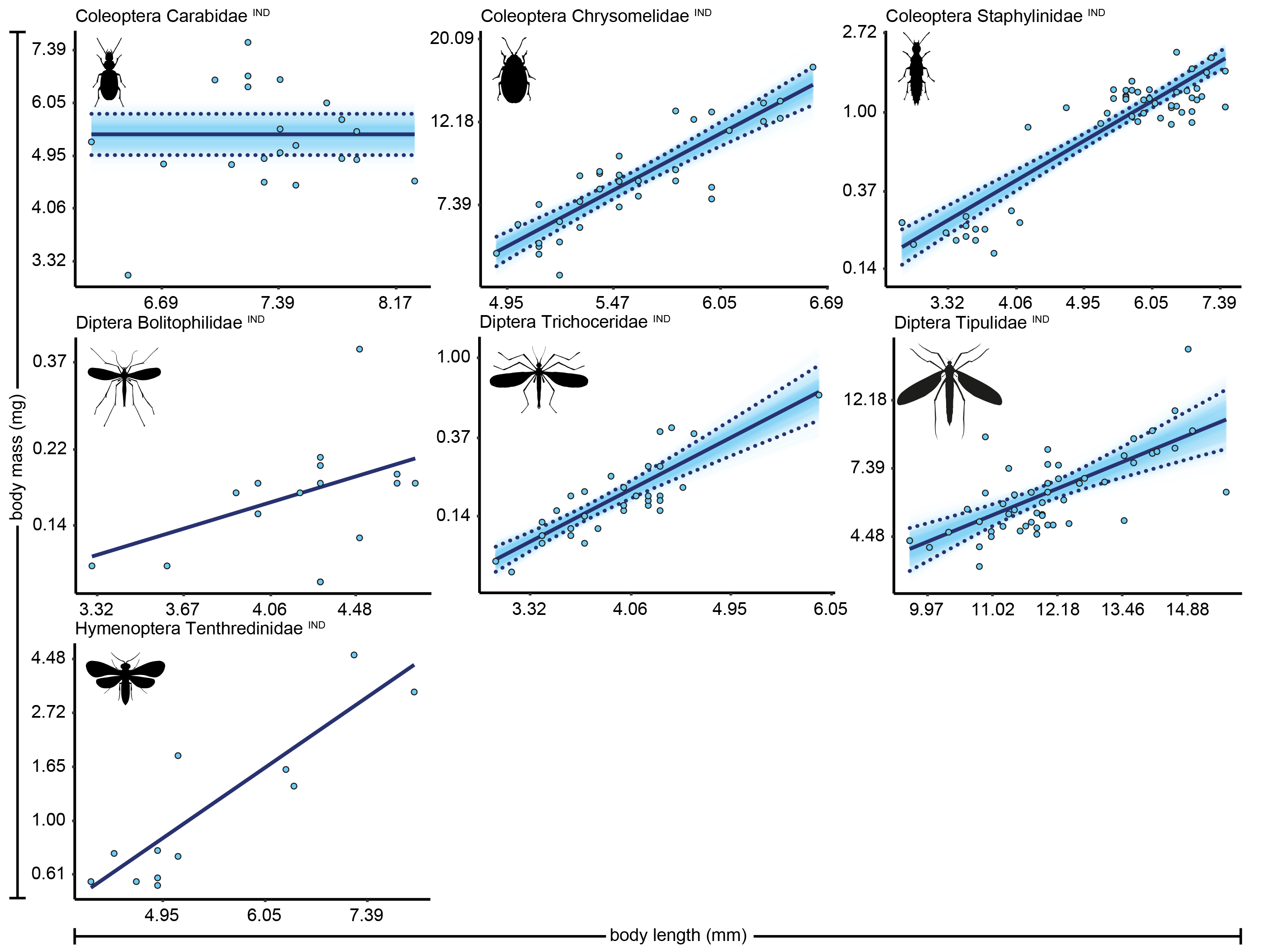

Supplement: Supplemental Information 3 — Axes are log-transformed but labelled with non-transformed values to ease interpretation. Superscripts following taxonomic names indicate whether datapoints represent individual level weight measurements (‘IND’) or averages per length group (‘AVG’). Raw datapoints are depicted as blue circles. Solid lines indicate the best supported model for each taxon. Dotted lines indicate 95% quantile confidence intervals calculated over 10,000 case bootstrapping runs. Individual bootstrapping runs are drawn as transparent lines to create a colour gradient that visualizes the distribution of best fitting models over all bootstrapping runs. [file peerj-11-15943-s003.png]

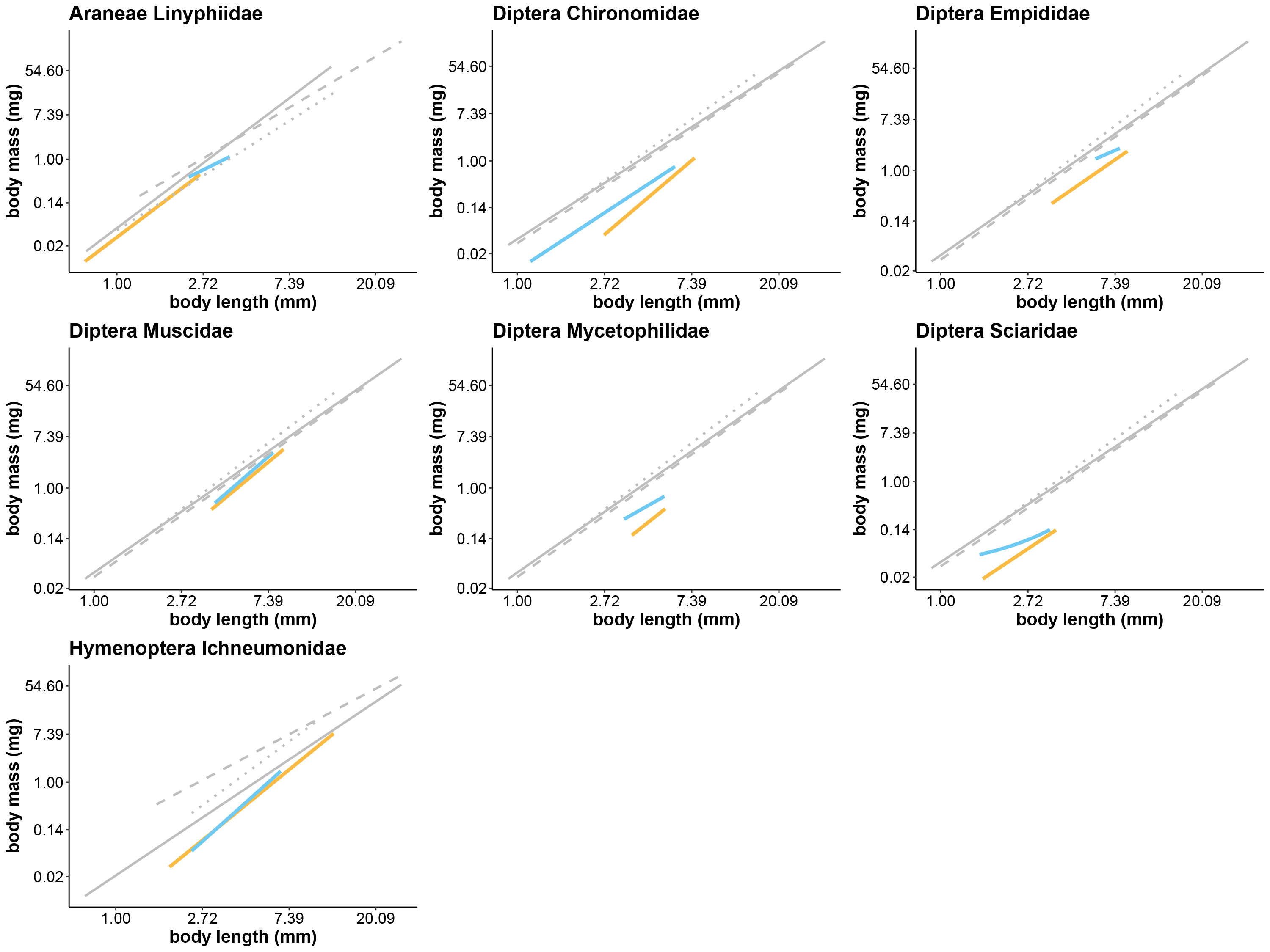

Supplement: Supplemental Information 4 — Data were available for both Zackenberg and Knipovich for these eight taxa only. Family-level regressions for Zackenberg are depicted in orange, while those for Knipovich are depicted in blue. Both are identical to the best supported models for each taxon plotted in Fig. 1. All other regressions depicted in grey are extracted from literature and correspond to order-level taxonomic resolution. Regressions depicted by solid grey lines are extracted from Rogers, Buschbom & Watson (1977), those by dashed grey lines from Hodar (1996) and those by dotted grey lines from Ganihar (1997). Axes are log-transformed but labelled with non-transformed values to ease interpretation. [file peerj-11-15943-s004.png]
