# Supplementary material for: Site-specific length-biomass relationships of arctic arthropod families are critical for accurate ecological inferences
Source: PeerJ. 2023 Sep 6;11:e15943. doi: 10.7717/peerj.15943 (PMC10492534; doi:10.7717/peerj.15943)
Supplement: Supplemental Information 5 — The column ‘Citations’ depicts the number of citations for each study extracted from Web of Science on 6 July 2023. All other columns indicate whether an allometric regression was available per taxonomic group, where each number indicates the sample size on which the regression was fit, and NA indicates that no regression was available. The three studies that we selected (based on being the most frequently cited and having regressions for all three taxonomic orders) are marked with an asterisk. Abbreviations: Ara, Araneae; Dip, Diptera; Hym, Hymenoptera; Lin, Linyphiidae; Chi, Chironomidae; Emp, Empididae; Mus, Muscidae; Myc, Mycetophilidae; Sci, Sciaridae; Ich, Ichneumonidae. [file peerj-11-15943-s005.docx]

| **Reference** | **Citations** | **Ara**  **Lin** | **Dip**  **Chi** | **Dip**  **Emp** | **Dip**  **Mus** | **Dip**  **Myc** | **Dip**  **Sci** | **Hym**  **Ich** | **Ara** | **Dip** | **Hym** |
| --- | --- | --- | --- | --- | --- | --- | --- | --- | --- | --- | --- |
| Sabo et al. 2002 | 204 | NA | NA | NA | NA | NA | NA | NA | NA | 61 | 54 |
| Hodar 1996* | 172 | NA | NA | NA | NA | NA | NA | NA | 18 | 36 | 24 |
| Rogers et al. 1977* | 171 | NA | NA | NA | NA | NA | NA | NA | 25 | 84 | 97 |
| Sample 1993 | 169 | NA | NA | NA | NA | NA | NA | 106 | NA | 257 | 274 |
| Schoener 1980 | 114 | NA | NA | NA | NA | NA | NA | NA | NA | 171 | 82 |
| Sage 1982 | 96 | NA | NA | NA | NA | NA | NA | NA | 39 | NA | NA |
| Ganihar 1997* | 83 | NA | NA | NA | NA | NA | NA | NA | 114 | 20 | 26 |
| Gowing and Recher 1984 | 53 | NA | NA | NA | NA | NA | NA | NA | 100 | 100 | 86 |
| Gruner 2003 | 51 | NA | NA | NA | NA | NA | NA | NA | 30 | 19 | 22 |
