# Supplementary material for: Site-specific length-biomass relationships of arctic arthropod families are critical for accurate ecological inferences
Source: PeerJ. 2023 Sep 6;11:e15943. doi: 10.7717/peerj.15943 (PMC10492534; doi:10.7717/peerj.15943)
Supplement: Supplemental Information 6 — The column ‘n’ depicts the number of data points on which each allometric model was fit with, if applicable, in brackets the sample size before averaging within different length classes. The length range of specimens used for fitting each regression is depicted by the columns ‘Min (mm)’ and ‘Max (mm)’. The columns ‘B0’, and ‘B1’ represent model parameters and, if applicable, in square brackets include 95% quantile confidence intervals calculated using non-parametric case-bootstrapping. The column ‘SF’ depicts a smearing factor that was calculated to correct back-transformed predictions for models with a log transformed response variable. The column ‘Level’ indicates whether the regression was fitted on individual level measurements or average values for different length classes. The column ‘Location’ indicates the site where arthropod specimens were collected. Abbreviations: ind, individual level weight measurements; avg, averaged weight estimates per length class; W, body mass (mg); SF, smearing factor; L, body length (mm); KNP, Knipovich; ZAC, Zackenberg. *Taxonomic level of Acari and Collembola is subclass. [file peerj-11-15943-s006.docx]

| **Order** | **Family** | **n** | **Min (mm)** | **Max (mm)** | **B0** | **B1** | **SF** | **Level** | **Model** | **Location** | |
| --- | --- | --- | --- | --- | --- | --- | --- | --- | --- | --- | --- |
| Acari* | *sp.* | 6 (605) | 0.29 | 1.64 | -3.627 | 2.012 | 1.028 | avg | ln(W/SF) = B0 + B1 * ln(L) | | ZAC |
| Acari* | *sp.* | 9 | 2.08 | 3.24 | -3.438 | 3.249 | 1.008 | ind | ln(W/SF) = B0 + B1 * ln(L) | | ZAC |
| Araneae | *Dictynidae* | 8 | 2.06 | 2.50 | -5.903 | 5.646 | 1.052 | ind | ln(W/SF) = B0 + B1 * ln(L) | | ZAC |
| Araneae | *Linyphiidae* | 28 | 2.30 | 3.70 | -2.422 [-2.968, -1.608] | 1.928 [1.202, 2.431] | 1.013 [1.007, 1.017] | ind | ln(W/SF) = B0 + B1 * ln(L) | | KNP |
| Araneae | *Linyphiidae* | 25 | 0.69 | 2.62 | -3.556 [-4.022, -2.867] | 2.938 [2.114, 3.464] | 1.035 [1.015, 1.053] | ind | ln(W/SF) = B0 + B1 * ln(L) | | ZAC |
| Araneae | *Lycosidae* | 129 | 1.74 | 8.68 | -3.718 [-4.087, -3.355] | 2.931 [2.742, 3.120] | 1.013 [1.009, 1.017] | ind | ln(W/SF) = B0 + B1 * ln(L) | | ZAC |
| Araneae | *Thomisidae* | 12 | 2.55 | 5.55 | -3.268 | 2.963 | 1.007 | ind | ln(W/SF) = B0 + B1 * ln(L) | | ZAC |
| Collembola* | *sp.* | 9 (209) | 0.65 | 2.56 | -0.015 | 0.026 | NA | avg | W = B0 + B1 * L | | KNP |
| Collembola* | *sp.* | 8 (1002) | 0.20 | 1.71 | -5.129 | 1.196 | 1.008 | avg | ln(W/SF) = B0 + B1 * ln(L) | | ZAC |
| Coleoptera | *Carabidae* | 21 | 6.30 | 8.30 | 5.371 [4.949, 5.802] | NA | NA | ind | W = B0 | | KNP |
| Coleoptera | *Chrysomelidae* | 34 | 4.90 | 6.60 | -3.672 [-4.622, -2.616] | 3.384 [2.765, 3.939] | 1.011 [1.006, 1.016] | ind | ln(W/SF) = B0 + B1 * ln(L) | | KNP |
| Coleoptera | *Staphylinidae* | 56 | 2.90 | 7.50 | -4.403 [-4.942, -3.851] | 2.498 [2.187, 2.813] | 1.051 [1.035, 1.064] | ind | ln(W/SF) = B0 + B1 * ln(L) | | KNP |
| Diptera | *Anthomyiidae* | 30 | 2.74 | 7.41 | -2.648 [-3.147, -2.105] | 0.510 [0.405, 0.597] | 1.039 [1.017, 1.062] | ind | ln(W/SF) = B0 + B1 * L | | ZAC |
| Diptera | *Bolitophilidae* | 16 | 3.30 | 4.80 | -4.061 | 1.559 | 1.05 | ind | ln(W/SF) = B0 + B1 * ln(L) | | KNP |
| Diptera | *Ceratopogonidae* | 37 (337) | 1.42 | 2.78 | -3.476 [-3.822, -3.207] | 0.765 [0.396, 1.213] | 1.019 [1.010, 1.028] | avg | ln(W/SF) = B0 + B1 * ln(L) | | ZAC |
| Diptera | *Chironomidae* | 83 | 1.16 | 6.10 | -4.738 [-4.958, -4.556] | 2.414 [2.171, 2.744] | 1.150 [1.103, 1.192] | ind | ln(W/SF) = B0 + B1 * ln(L) | | KNP |
| Diptera | *Chironomidae* | 67 (328) | 1.31 | 2.93 | -0.028 [-0.037, -0.018] | 0.031 [0.027, 0.036] | NA | avg | W = B0 + B1 * L | | ZAC |
| Diptera | *Chironomidae* | 45 | 2.70 | 7.64 | -6.286 [-7.578, -5.515] | 3.127 [2.498, 4.199] | 1.058 [1.017, 1.092] | ind | ln(W/SF) = B0 + B1 * ln(L) | | ZAC |
| Diptera | *Culicidae* | 78 | 3.97 | 6.94 | -3.093 [-4.184, -2.034] | 1.313 [0.693, 1.958] | 1.098 [1.068, 1.128] | ind | ln(W/SF) = B0 + B1 * ln(L) | | ZAC |
| Diptera | *Empididae* | 30 | 5.90 | 7.80 | -2.151 [-3.663, -0.465] | 1.467 [0.574, 2.264] | 1.011 [1.006, 1.016] | ind | ln(W/SF) = B0 + B1 * ln(L) | | KNP |
| Diptera | *Empididae* | 22 | 3.57 | 8.50 | -4.295 [-4.995, -0.375] | 2.350 [0.361, 2.741] | 1.025 [1.010, 1.038] | ind | ln(W/SF) = B0 + B1 * ln(L) | | ZAC |
| Diptera | *Muscidae* | 44 | 4.00 | 7.80 | -4.685 [-5.406, -4.127] | 2.949 [2.620, 3.373] | 1.013 [1.008, 1.018] | ind | ln(W/SF) = B0 + B1 * ln(L) | | KNP |
| Diptera | *Muscidae* | 412 | 3.84 | 8.80 | -4.679 [-4.858, -4.498] | 2.835 [2.733, 2.937] | 1.025 [1.022, 1.029] | ind | ln(W/SF) = B0 + B1 * ln(L) | | ZAC |
| Diptera | *Mycetophilidae* | 32 | 3.40 | 5.40 | -3.597 [-5.031, -0.859] | 1.923 [0.168, 2.859] | 1.031 [1.012, 1.046] | ind | ln(W/SF) = B0 + B1 * ln(L) | | KNP |
| Diptera | *Mycetophilidae* | 21 | 3.72 | 5.45 | -5.411 [-6.647, -4.085] | 2.701 [1.847, 3.516] | 1.022 [1.009, 1.034] | ind | ln(W/SF) = B0 + B1 * ln(L) | | ZAC |
| Diptera | *Phoridae* | 14 | 1.71 | 3.28 | -0.116 | 0.091 | NA | ind | W = B0 + B1 * L | | ZAC |
| Diptera | *Scathophagidae* | 35 | 5.45 | 8.93 | -3.717 [-4.641, -2.878] | 2.370 [1.948, 2.839] | 1.022 [1.012, 1.031] | ind | ln(W/SF) = B0 + B1 * ln(L) | | ZAC |
| Diptera | *Sciaridae* | 110 | 1.56 | 3.50 | -3.854 [-4.232, -3.452] | 0.525 [0.345, 0.685] | 1.047 [1.033, 1.063] | ind | ln(W/SF) = B0 + B1 * L | | KNP |
| Diptera | *Sciaridae* | 48 (273) | 1.62 | 3.75 | -5.164 [-5.570, -4.791] | 2.384 [1.996, 2.799] | 1.030 [1.016, 1.045] | avg | ln(W/SF) = B0 + B1 * ln(L) | | ZAC |
| Diptera | *Syrphidae* | 9 | 6.58 | 12.62 | -5.314 | 3.087 | 1.012 | ind | ln(W/SF) = B0 + B1 * ln(L) | | ZAC |
| Diptera | *Tachinidae* | 11 (28) | 10.56 | 12.48 | -3.952 | 2.617 | 1.005 | avg | ln(W/SF) = B0 + B1 * ln(L) | | ZAC |
| Diptera | *Tipulidae* | 52 | 9.70 | 15.8 | -3.033 [-4.844, -1.373] | 1.945 [1.266, 2.677] | 1.024 [1.012, 1.036] | ind | ln(W/SF) = B0 + B1 * ln(L) | | KNP |
| Diptera | *Trichoceridae* | 31 | 3.10 | 5.90 | -6.197 [-7.083, -5.208] | 3.234 [2.490, 3.907] | 1.035 [1.019, 1.047] | ind | ln(W/SF) = B0 + B1 * ln(L) | | KNP |
| Hymenoptera | *Ichneumonidae* | 22 | 2.40 | 6.7 | -5.739 [-6.510, -5.132] | 3.226 [2.767, 3.798] | 1.063 [1.024, 1.101] | ind | ln(W/SF) = B0 + B1 * ln(L) | | KNP |
| Hymenoptera | *Ichneumonidae* | 50 | 1.86 | 12.34 | -5.559 [-6.170, -4.862] | 2.928 [2.536, 3.273] | 1.254 [1.052, 1.497] | ind | ln(W/SF) = B0 + B1 * ln(L) | | ZAC |
| Hymenoptera | *Tenthredinidae* | 12 | 4.30 | 8.1 | -5.434 | 3.261 | 1.061 | ind | ln(W/SF) = B0 + B1 * ln(L) | | KNP |
| Lepidoptera | *Nymphalidae* | 61 | 12.34 | 15.09 | 3.408 [0.692, 6.069] | -0.330 [-1.342, 0.702] | 1.023 [1.012, 1.034] | ind | ln(W/SF) = B0 + B1 * ln(L) | | ZAC |
