# Supplementary material for: Site-specific length-biomass relationships of arctic arthropod families are critical for accurate ecological inferences
Source: PeerJ. 2023 Sep 6;11:e15943. doi: 10.7717/peerj.15943 (PMC10492534; doi:10.7717/peerj.15943)
Supplement: Supplemental Information 7 — Models marked in bold were selected as best supported model. This selection generally corresponded to the model with the lowest AIC score, but if multiple models were equally competitive (i.e., ΔAIC¡2 with the same number of estimated parameters) we preferred the power-model based on residual patterns and biological theoretic foundations. The column ‘n’ depicts the number of data points on which each allometric model was fit. The column ‘df’ indicates the number of estimated parameters in each model. The column ‘logLik’ depicts the log-likelihood of each model, and the column ‘AIC’ shows the AIC score for each model. The column ‘Level’ indicates whether the regression was fit on individual level measurements or average values for different length classes. The column ‘Location’ indicates the site where arthropod specimens were collected. Abbreviations: ind, individual level weight measurements; avg, averaged weight estimates per length class; W, body mass (mg); SF, smearing factor; L, body length (mm); KNP, Knipovich; ZAC, Zackenberg. [file peerj-11-15943-s007.docx]

| **Order** | **Family** | **n** | **df** | **logLik** | **AIC** | **Level** | **Model** | **Location** |
| --- | --- | --- | --- | --- | --- | --- | --- | --- |
| **Acari** | **sp.** | **6** | **3** | **0.155** | **-42.746** | **avg** | **ln(Weight/SF) = B0 + B1 * ln(length)** | **ZAC** |
| Acari | sp. | 6 | 3 | 23.151 | -40.302 | avg | Weight = B0 + B1 * length | ZAC |
| Acari | sp. | 6 | 3 | -4.195 | -34.045 | avg | ln(Weight/SF) = B0 + B1 * length | ZAC |
| Acari | sp. | 6 | 2 | 15.369 | -26.737 | avg | Weight = B0 | ZAC |
| Acari | sp. | 9 | 3 | 6.271 | -5.915 | ind | ln(Weight/SF) = B0 + B1 * length | ZAC |
| **Acari** | **sp.** | **9** | **3** | **5.920** | **-5.212** | **ind** | **ln(Weight/SF) = B0 + B1 * ln(length)** | **ZAC** |
| Acari | sp. | 9 | 3 | 4.718 | -3.436 | ind | Weight = B0 + B1 * length | ZAC |
| Acari | sp. | 9 | 2 | -4.737 | 13.475 | ind | Weight = B0 | ZAC |
| Araneae | Dictynidae | 8 | 3 | -2.118 | -10.780 | ind | ln(Weight/SF) = B0 + B1 * length | ZAC |
| **Araneae** | **Dictynidae** | **8** | **3** | **-2.219** | **-10.577** | **ind** | **ln(Weight/SF) = B0 + B1 * ln(length)** | **ZAC** |
| Araneae | Dictynidae | 8 | 3 | 4.203 | -2.406 | ind | Weight = B0 + B1 * length | ZAC |
| Araneae | Dictynidae | 8 | 2 | 1.454 | 1.093 | ind | Weight = B0 | ZAC |
| **Araneae** | **Linyphiidae** | **28** | **3** | **11.905** | **-33.916** | **ind** | **ln(Weight/SF) = B0 + B1 * ln(length)** | **KNP** |
| Araneae | Linyphiidae | 28 | 3 | 11.399 | -32.903 | ind | ln(Weight/SF) = B0 + B1 * length | KNP |
| Araneae | Linyphiidae | 28 | 3 | 17.181 | -28.363 | ind | Weight = B0 + B1 * length | KNP |
| Araneae | Linyphiidae | 28 | 2 | 5.674 | -7.349 | ind | Weight = B0 | KNP |
| **Araneae** | **Linyphiidae** | **25** | **3** | **-1.890** | **-61.322** | **ind** | **ln(Weight/SF) = B0 + B1 * ln(length)** | **ZAC** |
| Araneae | Linyphiidae | 25 | 3 | 32.213 | -58.426 | ind | Weight = B0 + B1 * length | ZAC |
| Araneae | Linyphiidae | 25 | 3 | -6.125 | -52.852 | ind | ln(Weight/SF) = B0 + B1 * length | ZAC |
| Araneae | Linyphiidae | 25 | 2 | 16.380 | -28.760 | ind | Weight = B0 | ZAC |
| **Araneae** | **Lycosidae** | **129** | **3** | **53.263** | **362.152** | **ind** | **ln(Weight/SF) = B0 + B1 * ln(length)** | **ZAC** |
| Araneae | Lycosidae | 129 | 3 | 18.818 | 431.042 | ind | ln(Weight/SF) = B0 + B1 * length | ZAC |
| Araneae | Lycosidae | 129 | 3 | -216.460 | 438.920 | ind | Weight = B0 + B1 * length | ZAC |
| Araneae | Lycosidae | 129 | 2 | -305.091 | 614.181 | ind | Weight = B0 | ZAC |
| Araneae | Thomisidae | 12 | 3 | 0.819 | 4.363 | ind | Weight = B0 + B1 * length | ZAC |
| **Araneae** | **Thomisidae** | **12** | **3** | **8.830** | **5.036** | **ind** | **ln(Weight/SF) = B0 + B1 * ln(length)** | **ZAC** |
| Araneae | Thomisidae | 12 | 3 | 4.433 | 13.830 | ind | ln(Weight/SF) = B0 + B1 * length | ZAC |
| Araneae | Thomisidae | 12 | 2 | -19.365 | 42.730 | ind | Weight = B0 | ZAC |
| **Coleoptera** | **Carabidae** | **21** | **2** | **-29.514** | **63.027** | **ind** | **Weight = B0** | **KNP** |
| Coleoptera | Carabidae | 21 | 3 | -29.488 | 64.975 | ind | Weight = B0 + B1 * length | KNP |
| Coleoptera | Carabidae | 21 | 3 | 5.427 | 65.024 | ind | ln(Weight/SF) = B0 + B1 * ln(length) | KNP |
| Coleoptera | Carabidae | 21 | 3 | 5.381 | 65.116 | ind | ln(Weight/SF) = B0 + B1 * length | KNP |
| **Coleoptera** | **Chrysomelidae** | **34** | **3** | **15.906** | **120.393** | **ind** | **ln(Weight/SF) = B0 + B1 * ln(length)** | **KNP** |
| Coleoptera | Chrysomelidae | 34 | 3 | 15.769 | 120.667 | ind | ln(Weight/SF) = B0 + B1 * length | KNP |
| Coleoptera | Chrysomelidae | 34 | 3 | -59.525 | 125.049 | ind | Weight = B0 + B1 * length | KNP |
| Coleoptera | Chrysomelidae | 34 | 2 | -84.294 | 172.587 | ind | Weight = B0 | KNP |
| **Coleoptera** | **Staphylinidae** | **56** | **3** | **-14.734** | **8.627** | **ind** | **ln(Weight/SF) = B0 + B1 * ln(length)** | **KNP** |
| Coleoptera | Staphylinidae | 56 | 3 | -5.483 | 16.967 | ind | Weight = B0 + B1 * length | KNP |
| Coleoptera | Staphylinidae | 56 | 3 | -20.375 | 19.909 | ind | ln(Weight/SF) = B0 + B1 * length | KNP |
| Coleoptera | Staphylinidae | 56 | 2 | -40.183 | 84.365 | ind | Weight = B0 | KNP |
| **Collembola** | **sp.** | **9** | **3** | **42.927** | **-79.854** | **avg** | **Weight = B0 + B1 * length** | **KNP** |
| Collembola | sp. | 9 | 3 | 3.697 | -72.405 | avg | ln(Weight/SF) = B0 + B1 * ln(length) | KNP |
| Collembola | sp. | 9 | 3 | -2.134 | -60.743 | avg | ln(Weight/SF) = B0 + B1 * length | KNP |
| Collembola | sp. | 9 | 2 | 24.247 | -44.494 | avg | Weight = B0 | KNP |
| **Collembola** | **sp.** | **8** | **3** | **5.226** | **-92.032** | **avg** | **ln(Weight/SF) = B0 + B1 * ln(length)** | **ZAC** |
| Collembola | sp. | 8 | 3 | 48.657 | -91.314 | avg | Weight = B0 + B1 * length | ZAC |
| Collembola | sp. | 8 | 3 | -1.540 | -78.499 | avg | ln(Weight/SF) = B0 + B1 * length | ZAC |
| Collembola | sp. | 8 | 2 | 33.828 | -63.655 | avg | Weight = B0 | ZAC |
| **Diptera** | **Anthomyiidae** | **30** | **3** | **-5.605** | **24.705** | **ind** | **ln(Weight/SF) = B0 + B1 * length** | **ZAC** |
| Diptera | Anthomyiidae | 30 | 3 | -7.167 | 27.828 | ind | ln(Weight/SF) = B0 + B1 * ln(length) | ZAC |
| Diptera | Anthomyiidae | 30 | 3 | -20.179 | 46.359 | ind | Weight = B0 + B1 * length | ZAC |
| Diptera | Anthomyiidae | 30 | 2 | -36.853 | 77.706 | ind | Weight = B0 | ZAC |
| **Diptera** | **Bolitophilidae** | **16** | **3** | **-3.573** | **-45.068** | **ind** | **ln(Weight/SF) = B0 + B1 * ln(length)** | **KNP** |
| Diptera | Bolitophilidae | 16 | 3 | -3.608 | -44.998 | ind | ln(Weight/SF) = B0 + B1 * length | KNP |
| Diptera | Bolitophilidae | 16 | 3 | 21.453 | -36.905 | ind | Weight = B0 + B1 * length | KNP |
| Diptera | Bolitophilidae | 16 | 2 | 20.189 | -36.378 | ind | Weight = B0 | KNP |
| Diptera | Ceratopogonidae | 37 | 3 | 116.997 | -227.994 | avg | Weight = B0 + B1 * length | ZAC |
| Diptera | Ceratopogonidae | 37 | 3 | 7.989 | -227.752 | avg | ln(Weight/SF) = B0 + B1 * length | ZAC |
| **Diptera** | **Ceratopogonidae** | **37** | **3** | **7.471** | **-226.717** | **avg** | **ln(Weight/SF) = B0 + B1 * ln(length)** | **ZAC** |
| Diptera | Ceratopogonidae | 37 | 2 | 109.190 | -214.381 | avg | Weight = B0 | ZAC |
| **Diptera** | **Chironomidae** | **83** | **3** | **-62.704** | **-395.525** | **ind** | **ln(Weight/SF) = B0 + B1 * ln(length)** | **KNP** |
| Diptera | Chironomidae | 83 | 3 | -72.143 | -376.647 | ind | ln(Weight/SF) = B0 + B1 * length | KNP |
| Diptera | Chironomidae | 83 | 3 | 134.368 | -262.737 | ind | Weight = B0 + B1 * length | KNP |
| Diptera | Chironomidae | 83 | 2 | 89.769 | -175.537 | ind | Weight = B0 | KNP |
| **Diptera** | **Chironomidae** | **67** | **3** | **217.704** | **-429.408** | **avg** | **Weight = B0 + B1 * length** | **ZAC** |
| Diptera | Chironomidae | 67 | 3 | -15.192 | -418.686 | avg | ln(Weight/SF) = B0 + B1 * ln(length) | ZAC |
| Diptera | Chironomidae | 67 | 3 | -17.342 | -414.387 | avg | ln(Weight/SF) = B0 + B1 * length | ZAC |
| Diptera | Chironomidae | 67 | 2 | 176.776 | -349.551 | avg | Weight = B0 | ZAC |
| **Diptera** | **Chironomidae** | **45** | **3** | **-11.224** | **-183.478** | **ind** | **ln(Weight/SF) = B0 + B1 * ln(length)** | **ZAC** |
| Diptera | Chironomidae | 45 | 3 | -16.205 | -173.514 | ind | ln(Weight/SF) = B0 + B1 * length | ZAC |
| Diptera | Chironomidae | 45 | 3 | 33.810 | -61.621 | ind | Weight = B0 + B1 * length | ZAC |
| Diptera | Chironomidae | 45 | 2 | 13.178 | -22.357 | ind | Weight = B0 | ZAC |
| **Diptera** | **Culicidae** | **78** | **3** | **-43.819** | **-46.284** | **ind** | **ln(Weight/SF) = B0 + B1 * ln(length)** | **ZAC** |
| Diptera | Culicidae | 78 | 3 | -44.341 | -45.240 | ind | ln(Weight/SF) = B0 + B1 * length | ZAC |
| Diptera | Culicidae | 78 | 3 | 12.576 | -19.153 | ind | Weight = B0 + B1 * length | ZAC |
| Diptera | Culicidae | 78 | 2 | 9.210 | -14.420 | ind | Weight = B0 | ZAC |
| Diptera | Empididae | 30 | 3 | 14.898 | 14.560 | ind | ln(Weight/SF) = B0 + B1 * length | KNP |
| **Diptera** | **Empididae** | **30** | **3** | **14.623** | **15.111** | **ind** | **ln(Weight/SF) = B0 + B1 * ln(length)** | **KNP** |
| Diptera | Empididae | 30 | 3 | -7.227 | 20.453 | ind | Weight = B0 + B1 * length | KNP |
| Diptera | Empididae | 30 | 2 | -13.577 | 31.153 | ind | Weight = B0 | KNP |
| Diptera | Empididae | 22 | 3 | -1.900 | 9.799 | ind | Weight = B0 + B1 * length | ZAC |
| **Diptera** | **Empididae** | **22** | **3** | **1.732** | **10.367** | **ind** | **ln(Weight/SF) = B0 + B1 * ln(length)** | **ZAC** |
| Diptera | Empididae | 22 | 3 | -1.753 | 17.338 | ind | ln(Weight/SF) = B0 + B1 * length | ZAC |
| Diptera | Empididae | 22 | 2 | -12.229 | 28.457 | ind | Weight = B0 | ZAC |
| **Diptera** | **Muscidae** | **44** | **3** | **17.467** | **3.390** | **ind** | **ln(Weight/SF) = B0 + B1 * ln(length)** | **KNP** |
| Diptera | Muscidae | 44 | 3 | 15.545 | 7.234 | ind | ln(Weight/SF) = B0 + B1 * length | KNP |
| Diptera | Muscidae | 44 | 3 | -16.393 | 38.787 | ind | Weight = B0 + B1 * length | KNP |
| Diptera | Muscidae | 44 | 2 | -55.825 | 115.649 | ind | Weight = B0 | KNP |
| **Diptera** | **Muscidae** | **412** | **3** | **35.168** | **276.004** | **ind** | **ln(Weight/SF) = B0 + B1 * ln(length)** | **ZAC** |
| Diptera | Muscidae | 412 | 3 | 21.845 | 302.651 | ind | ln(Weight/SF) = B0 + B1 * length | ZAC |
| Diptera | Muscidae | 412 | 3 | -267.639 | 541.279 | ind | Weight = B0 + B1 * length | ZAC |
| Diptera | Muscidae | 412 | 2 | -543.813 | 1091.625 | ind | Weight = B0 | ZAC |
| **Diptera** | **Mycetophilidae** | **32** | **3** | **0.154** | **-36.849** | **ind** | **ln(Weight/SF) = B0 + B1 * ln(length)** | **KNP** |
| Diptera | Mycetophilidae | 32 | 3 | -0.669 | -35.203 | ind | ln(Weight/SF) = B0 + B1 * length | KNP |
| Diptera | Mycetophilidae | 32 | 3 | 20.313 | -34.626 | ind | Weight = B0 + B1 * length | KNP |
| Diptera | Mycetophilidae | 32 | 2 | 16.138 | -28.275 | ind | Weight = B0 | KNP |
| **Diptera** | **Mycetophilidae** | **21** | **3** | **3.498** | **-55.602** | **ind** | **ln(Weight/SF) = B0 + B1 * ln(length)** | **ZAC** |
| Diptera | Mycetophilidae | 21 | 3 | 3.157 | -54.921 | ind | ln(Weight/SF) = B0 + B1 * length | ZAC |
| Diptera | Mycetophilidae | 21 | 3 | 28.245 | -50.491 | ind | Weight = B0 + B1 * length | ZAC |
| Diptera | Mycetophilidae | 21 | 2 | 19.980 | -35.960 | ind | Weight = B0 | ZAC |
| **Diptera** | **Phoridae** | **14** | **3** | **43.617** | **-81.233** | **ind** | **Weight = B0 + B1 * length** | **ZAC** |
| Diptera | Phoridae | 14 | 3 | 2.307 | -76.059 | ind | ln(Weight/SF) = B0 + B1 * ln(length) | ZAC |
| Diptera | Phoridae | 14 | 3 | 1.455 | -74.354 | ind | ln(Weight/SF) = B0 + B1 * length | ZAC |
| Diptera | Phoridae | 14 | 2 | 25.754 | -47.508 | ind | Weight = B0 | ZAC |
| Diptera | Scathophagidae | 35 | 3 | 4.987 | 54.201 | ind | ln(Weight/SF) = B0 + B1 * length | ZAC |
| **Diptera** | **Scathophagidae** | **35** | **3** | **4.893** | **54.388** | **ind** | **ln(Weight/SF) = B0 + B1 * ln(length)** | **ZAC** |
| Diptera | Scathophagidae | 35 | 3 | -26.074 | 58.149 | ind | Weight = B0 + B1 * length | ZAC |
| Diptera | Scathophagidae | 35 | 2 | -49.278 | 102.556 | ind | Weight = B0 | ZAC |
| **Diptera** | **Sciaridae** | **110** | **3** | **-28.516** | **-515.981** | **ind** | **ln(Weight/SF) = B0 + B1 * length** | **KNP** |
| Diptera | Sciaridae | 110 | 3 | -30.999 | -511.015 | ind | ln(Weight/SF) = B0 + B1 * ln(length) | KNP |
| Diptera | Sciaridae | 110 | 3 | 248.534 | -491.067 | ind | Weight = B0 + B1 * length | KNP |
| Diptera | Sciaridae | 110 | 2 | 223.283 | -442.566 | ind | Weight = B0 | KNP |
| **Diptera** | **Sciaridae** | **48** | **3** | **-2.006** | **-280.922** | **avg** | **ln(Weight/SF) = B0 + B1 * ln(length)** | **ZAC** |
| Diptera | Sciaridae | 48 | 3 | -2.791 | -279.352 | avg | ln(Weight/SF) = B0 + B1 * length | ZAC |
| Diptera | Sciaridae | 48 | 3 | 129.493 | -252.985 | avg | Weight = B0 + B1 * length | ZAC |
| Diptera | Sciaridae | 48 | 2 | 92.578 | -181.156 | avg | Weight = B0 | ZAC |
| **Diptera** | **Syrphidae** | **9** | **3** | **4.227** | **21.283** | **ind** | **ln(Weight/SF) = B0 + B1 * ln(length)** | **ZAC** |
| Diptera | Syrphidae | 9 | 3 | 3.240 | 23.258 | ind | ln(Weight/SF) = B0 + B1 * length | ZAC |
| Diptera | Syrphidae | 9 | 3 | -11.500 | 29.000 | ind | Weight = B0 + B1 * length | ZAC |
| Diptera | Syrphidae | 9 | 2 | -24.852 | 53.703 | ind | Weight = B0 | ZAC |
| Diptera | Tachinidae | 11 | 3 | 9.526 | 40.882 | avg | ln(Weight/SF) = B0 + B1 * length | ZAC |
| **Diptera** | **Tachinidae** | **11** | **3** | **9.470** | **40.993** | **avg** | **ln(Weight/SF) = B0 + B1 * ln(length)** | **ZAC** |
| Diptera | Tachinidae | 11 | 3 | -18.299 | 42.598 | avg | Weight = B0 + B1 * length | ZAC |
| Diptera | Tachinidae | 11 | 2 | -23.351 | 50.703 | avg | Weight = B0 | ZAC |
| Diptera | Tipulidae | 52 | 3 | 6.949 | 181.089 | ind | ln(Weight/SF) = B0 + B1 * length | KNP |
| **Diptera** | **Tipulidae** | **52** | **3** | **6.859** | **181.270** | **ind** | **ln(Weight/SF) = B0 + B1 * ln(length)** | **KNP** |
| Diptera | Tipulidae | 52 | 3 | -102.319 | 210.637 | ind | Weight = B0 + B1 * length | KNP |
| Diptera | Tipulidae | 52 | 2 | -117.763 | 239.526 | ind | Weight = B0 | KNP |
| **Diptera** | **Trichoceridae** | **31** | **3** | **-1.856** | **-99.172** | **ind** | **ln(Weight/SF) = B0 + B1 * ln(length)** | **KNP** |
| Diptera | Trichoceridae | 31 | 3 | -2.267 | -98.350 | ind | ln(Weight/SF) = B0 + B1 * length | KNP |
| Diptera | Trichoceridae | 31 | 3 | 41.458 | -76.915 | ind | Weight = B0 + B1 * length | KNP |
| Diptera | Trichoceridae | 31 | 2 | 22.682 | -41.364 | ind | Weight = B0 | KNP |
| **Hymenoptera** | **Ichneumonidae** | **22** | **3** | **-7.329** | **-51.531** | **ind** | **ln(Weight/SF) = B0 + B1 * ln(length)** | **KNP** |
| Hymenoptera | Ichneumonidae | 22 | 3 | -8.281 | -49.627 | ind | ln(Weight/SF) = B0 + B1 * length | KNP |
| Hymenoptera | Ichneumonidae | 22 | 3 | 12.081 | -18.162 | ind | Weight = B0 + B1 * length | KNP |
| Hymenoptera | Ichneumonidae | 22 | 2 | -10.494 | 24.987 | ind | Weight = B0 | KNP |
| **Hymenoptera** | **Ichneumonidae** | **50** | **3** | **-46.965** | **-9.878** | **ind** | **ln(Weight/SF) = B0 + B1 * ln(length)** | **ZAC** |
| Hymenoptera | Ichneumonidae | 50 | 3 | -51.817 | -0.173 | ind | ln(Weight/SF) = B0 + B1 * length | ZAC |
| Hymenoptera | Ichneumonidae | 50 | 3 | -57.169 | 120.338 | ind | Weight = B0 + B1 * length | ZAC |
| Hymenoptera | Ichneumonidae | 50 | 2 | -92.948 | 189.896 | ind | Weight = B0 | ZAC |
| **Hymenoptera** | **Tenthredinidae** | **12** | **3** | **-3.602** | **15.163** | **ind** | **ln(Weight/SF) = B0 + B1 * ln(length)** | **KNP** |
| Hymenoptera | Tenthredinidae | 12 | 3 | -3.657 | 15.274 | ind | ln(Weight/SF) = B0 + B1 * length | KNP |
| Hymenoptera | Tenthredinidae | 12 | 3 | -11.926 | 29.852 | ind | Weight = B0 + B1 * length | KNP |
| Hymenoptera | Tenthredinidae | 12 | 2 | -19.607 | 43.215 | ind | Weight = B0 | KNP |
| **Lepidoptera** | **Nymphalidae** | **61** | **3** | **10.275** | **295.749** | **ind** | **ln(Weight/SF) = B0 + B1 * ln(length)** | **ZAC** |
| Lepidoptera | Nymphalidae | 61 | 3 | 10.275 | 295.749 | ind | ln(Weight/SF) = B0 + B1 * length | ZAC |
| Lepidoptera | Nymphalidae | 61 | 2 | -154.053 | 312.106 | ind | Weight = B0 | ZAC |
| Lepidoptera | Nymphalidae | 61 | 3 | -153.823 | 313.646 | ind | Weight = B0 + B1 * length | ZAC |
